# Novel Technique in Performing Ocular Ultrasound in Trauma: A Case Series

**DOI:** 10.5811/cpcem.48653

**Published:** 2026-06-25

**Authors:** Hannah Chawang, Sanjeev Bhoi, Vineeth Chandran, Anmol Chanda, Anand Kumar Das

**Affiliations:** JPN Apex Trauma Centre, Department of Emergency Medicine, All India Institute of Medical Sciences, New Delhi, India

**Keywords:** modified water bath technique, ocular ultrasound, trauma, facial injuries, case series

## Abstract

**Introduction:**

Ocular ultrasound is highly effective for diagnosing traumatic eye injuries such as retinal detachment, vitreous hemorrhage, foreign bodies, and retrobulbar hematomas. However, it is contraindicated in cases of suspected globe rupture, as applying external pressure to the eye could cause further damage. When globe injury is clinically suspected, computed tomography is typically used to confirm the diagnosis. However, delays can occur if other life-threatening injuries require immediate attention or there are long wait times. We propose a novel method for performing ocular ultrasound in such cases—the modified water bath technique—which allows for the diagnosis of globe rupture upon the patient’s arrival in the emergency department.

**Case Series:**

We present five cases where the modified water bath technique was used. A glove partially filled with saline is prepared and gently placed on the affected eye while the patient lies supine. A small amount of gel is applied to a high-frequency linear probe, which is then positioned transversely on the glove. An ocular scan is then conducted in dual mode to compare both eyes.

**Conclusion:**

This method produces clear, well-defined images and offers the advantage of being performed bedside immediately. This eliminates diagnostic delays and allows for prompt initiation of treatment. Additionally, the high-quality images provided by this technique can be used to diagnose other eye emergencies through ultrasound.

## INTRODUCTION

Nearly 60 million cases of ocular trauma were reported worldwide in 2019, resulting in over 400,000 years lived with disability, according to the Global Burden of Disease Study, Eye injuries are a major cause of visual impairment, with 1.6 million cases of blindness globally attributed to ocular trauma.^1^ Unintentional injuries such as intraocular foreign bodies, mechanical forces, and falls were the leading causes, followed by self-harm, interpersonal violence, and transport-related injuries.^2^ Vision-threatening injuries, such as open globe injuries, vitreous hemorrhage, retinal detachment, and retrobulbar hematoma, require prompt recognition and early ophthalmologic intervention to optimize visual outcomes. About 27% of serious ocular injuries in the United States result in legal blindness (visual acuity worse than 20/200 where normal is considered 20/20).^3^

Ocular ultrasound is a vital diagnostic tool in the emergency department (ED), particularly for assessing traumatic eye injuries. Common pathologies identified via this modality include retinal detachment, vitreous haemorrhage, intraocular foreign bodies, and retrobulbar hematomas. It has been useful when physical examination has proven to be inadequate, especially when the visual axis is obstructed due to foreign body or blood. Even with extensive facial swelling, lid manipulation will be painful and may even cause more harm if there is globe rupture. Ultrasound allows for visualisation of the retina without dilating the pupil.^4^

Despite its utility, ocular ultrasound also presents distinct challenges. In cases of suspected globe rupture, without proper precautions even minimal pressure on the eyelid can exacerbate the injury, increasing the risk of vitreous extrusion. Additionally, the procedure can be intensely painful and uncomfortable for the patient, further complicating its execution. Another common issue is the difficulty in visualising near-field structures, which are often positioned too close to the transducer, resulting in suboptimal imaging.

In such scenarios, the diagnostic gold standard has been computed tomography (CT) for globe rupture, presence of foreign body, retrobulbar hematoma, etc.^5^ However, reliance on CT can lead to delays, particularly when resources are limited or other critical injuries take precedence. To address these challenges, we propose the modified water bath technique for ocular ultrasound. This technique is an adaptation of the water bath technique used for evaluating injuries to extremities, such as fractures in the hand or foot. By using water as a medium to optimize image clarity in those cases, this method significantly improves the visualisation and interpretation of fractures.^6^

This novel approach to ocular ultrasound offers a non-invasive, bedside diagnostic option that produces high-quality images without compromising patient safety or comfort. In this paper we detail the methodology and discuss the clinical advantages of this innovative technique.

## CASE SERIES

We report five cases in which the modified water bath technique was used to perform ocular ultrasound in the ED of a trauma centre. Every patient was initially managed by performing the primary and secondary survey. Once adequate resuscitation had been confirmed and the patient was hemodynamically stable, ultrasound was done in those patients with suspected ocular injuries to help guide further treatment.

*CPC-EM Capsule*

What do we already know about this clinical entity?

*Ocular ultrasound is a valuable diagnostic tool for eye injuries, but direct probe contact may cause discomfort and risk further increase in intraocular pressure, especially in patients with traumatic eye injuries.*

What makes this presentation of disease reportable?

*This report describes a modified water bath technique which is a novel technique in performing ocular ultrasound that enables a safer, more comfortable method with better quality images when compared to the conventional method.*

What is the major learning point?

*The modified water bath technique permits high-quality ocular ultrasound images while minimising patient discomfort and avoiding an increase in globe pressure.*

How might this improve emergency medicine practice?

*This technique expands safe ocular ultrasound use in emergency settings, particularly for trauma or painful eye conditions, while providing superior image quality.*

### Modified Water Bath Technique

**Image 1:**
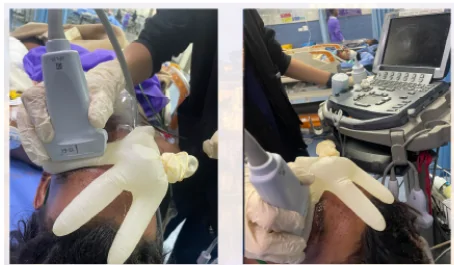
Modified water bath technique in a case series describing its use to treat ocular trauma in the emergency department.

**Image 2:**
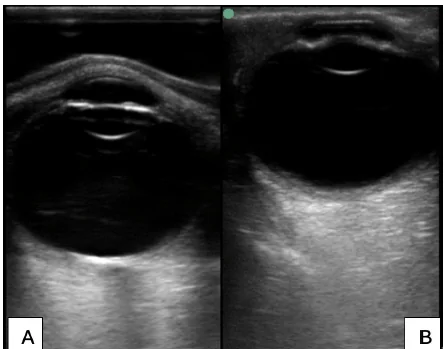
Ocular ultrasound performed using (A) modified water bath* technique and (B) conventional method. *Saline solution was used as the medium.

1. **Preparation:**
  - Partially fill a sterile surgical glove with saline solution and secure it to prevent leakage.
  - Gently place the glove over the closed eyelid of the affected eye while the patient remains in a supine position, as shown in Image 1.
2. **Ultrasound Setup:**
  - Prepare to position a high-frequency 6–13 megahertz linear transducer by gently placing saline-soaked cotton on the surface, ensuring optimal conductivity and reducing artifacts during imaging.
  - Select ocular preset, and keep the thermal index < 1 and spatial-peak temporal-average < 50 milliwatts per square centimeter, per the U.S. Food and Drug administration guidelines for ophthalmic ultrasound manufacturers.^7^
  - Position the transducer transversely on the saline-filled glove and lay it on one of the gloved fingers, avoiding direct contact with the eyelid or globe.
3. **Imaging Protocol:**
  - We used a dual-mode imaging protocol, enabling simultaneous comparison of the affected eye with the unaffected eye.
  - Images were captured systematically, covering all relevant ocular structures to ensure a comprehensive evaluation. Image 2 shows the dual-mode imaging of ocular ultrasound performed on a patient using both the modified water bath technique and the conventional method.

Ocular ultrasound has been pivotal in diagnosing vision-threatening injuries. However, it has several limitations in trauma cases. Firstly, uncooperative patients often complicate probe placement as the procedure causes discomfort. Secondly, in patients with suspected globe rupture, a generous amount of gel must be applied over the eyelid to minimize the risk of vitreous extrusion caused by excessive pressure. Therefore, the skill of the operator in applying minimal pressure, whilst also acquiring good-quality image, plays a critical role. Finally, technical issues, which include difficulty visualising near-field structures as well as peripheral structures, may lead to difficulty in acquiring and optimizing the image.

Ocular ultrasound was performed by an emergency medicine senior resident. Patient comfort and procedural feasibility were evaluated through patient verbal feedback and clinician observations.

## DISCUSSION

The National Trauma Data Bank (2008–2014) showed that 58.2% of patients with major trauma and ocular injuries had traumatic brain injuries and were often associated with facial fractures (61.3%).^8^ This leads to challenges while examining patients initially. Extensive periorbital swelling makes lid manipulation difficult, and a patient with altered mentation will not be as compliant with an eye examination. It can also be difficult to examine the eye when foreign bodies or blood obscures the visual axis.^4^ Additionally, life-threatening conditions take priority, which may delay the identification of ocular trauma.

Addressing these limitations, we propose the use of a modified water bath technique for identifying ocular trauma, which is a modification of the water bath technique already in use to evaluate extremity injuries. Shrimal and the study group of our institute studied this technique by immersing the affected limb in a basin filled with lukewarm water. The ultrasound transducer was positioned near the water’s surface without making direct contact with the skin. The quality of images significantly improved when water was used as a medium compared to the conventional gel. This further improved the visualisation and interpretation of fracture and showed a sensitivity and specificity of 97% and 94%, respectively, for identifying fractures of the. hand and foot.^6^ Using the same concept, we developed the modified water bath technique for ocular ultrasound.

Table 1 summarises the five cases with patient details, the mechanism of injury, final diagnosis, ultrasound findings using the modified water bath technique and the findings on CT. The modified water bath technique consistently produced clear, well-defined ocular images. These high-quality images were instrumental in identifying globe rupture, foreign body, and vitreous haemorrhage with diagnostic precision. The anterior chamber, iris, lens, and vitreous chamber were all visualised well. The near-field structures as well as peripheral ocular structures were clearly visualised. The conventional method resulted in lower image quality as the patient was uncomfortable due to pain, which prevented full placement of the ultrasound probe on the eyelid.

After viewing the images in ultrasound using the modified water bath technique, we correlated the images with head CT. Computed tomography is considered the gold standard in trauma because it is useful for both diagnosing facial fractures and evaluating orbital soft tissue injuries and foreign body. It has a higher specificity (90–100%) for identifying open globe injuries where most specific CT indicators are alterations in the globe and presence of vitreous haemorrhage (specificity 98%).^9^ Computed tomography can detect intraocular foreign body as small as 0.06 mm^3^ with a sensitivity exceeding 65% and specificity of 90%.^10^,^11^ However, detection rates are based on the material of the foreign body. Also, subtle soft tissue injuries can be missed, which is why CT is never used as the sole imaging modality.

Ultrasound should complement examination. as soft tissue injuries such as vitreous haemorrhage, retinal detachment, choroid detachment, lens dislocation, etc, are better visualised with a sensitivity of approximately 93% and a specificity of 98%.^12^ Ocular ultrasound compares favourably with CT in all measures of accuracy.^13^ Since the image quality using the modified water bath technique supersedes the quality of conventional ocular ultrasound imaging, we infer that the correlation between images obtained using the water bath technique and images seen in the CT were better as well, as seen in all five cases.

This technique is also very straightforward, requiring minimal additional equipment beyond standard ultrasound tools. The simplicity of the method makes it highly adaptable for emergency settings, where time and resources are often limited. Feedback from patients indicated a high level of comfort during the procedure. The use of a saline-filled glove, rather than direct application of the probe, reduced the sensation of pressure on the eye, alleviating patient anxiety and minimizing the risk of exacerbating the injury. Table 2 summarises the benefits of this novel technique.

While the results of this small case series are promising, larger scale studies are needed to validate the technique’s efficacy across a broader patient population. Future research could also explore adaptations of this method for other ocular conditions and further refine the imaging protocol.

## CONCLUSION

The modified water bath technique provides clear, high-quality ocular images, enhancing diagnostic accuracy while minimizing patient discomfort. Its simplicity and minimal equipment requirements make it well-suited for emergency settings. However, larger studies are needed to validate its efficacy and explore broader applications.

## Figures and Tables

**Image 3:**
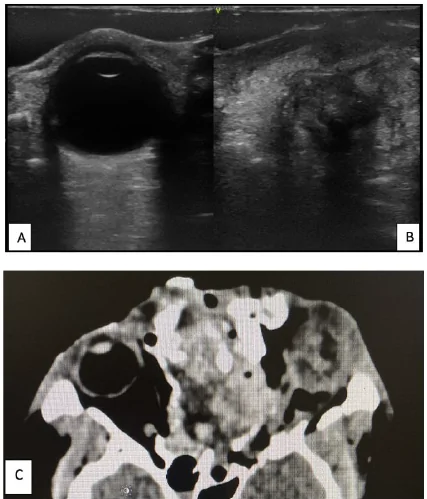
Case 1. Modified water bath technique used in a patient globe rupture: A) normal right eye; B) globe rupture of left eye; and C) computed tomography suggestive of left globe rupture.

**Image 4:**
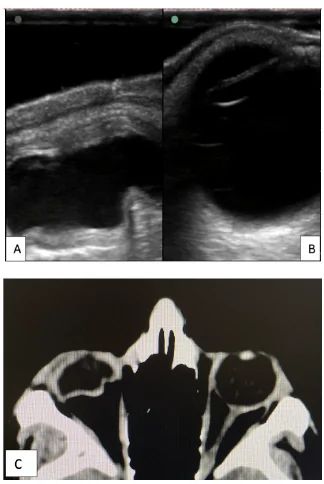
Case 2. Ocular ultrasound modified water bath technique performed: A) right globe injury with loss of globe volume and decrease in size; B) normal left eye; and C) computed tomography findings suggestive of right globe injury.

**Image 5:**
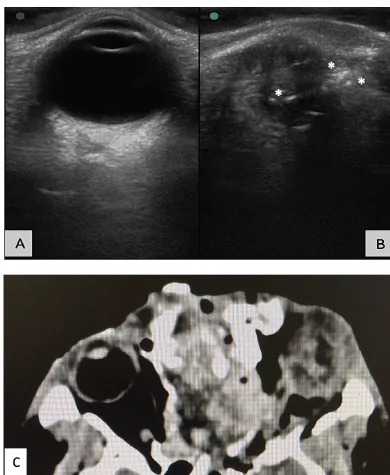
Case 3. Ocular ultrasound modified water bath technique performed: A) right normal eye; B) left globe rupture with intraocular foreign body seen as echogenic structures with posterior acoustic shadowing (marked with asterisks); and C) computed tomography suggestive of left globe rupture.

**Image 6:**
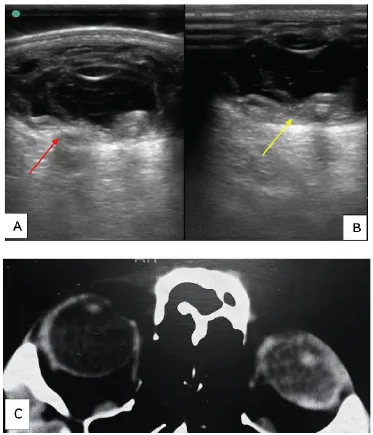
Case 4. A patient with suspected vitreous haemorrhage and globe rupture: A) using modified water bath technique (red arrow); B) using conventional method on the same eye using copious amount of gel (yellow arrow); and C) computed tomography suggestive of left globe injury.

**Image 7:**
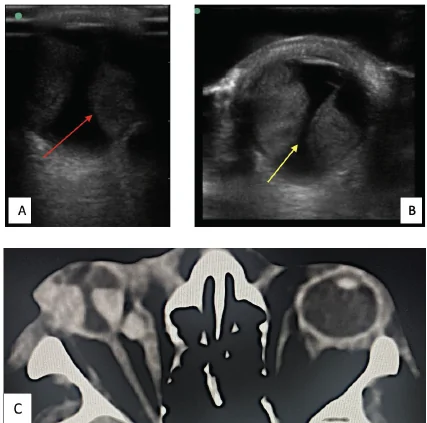
Case 5. A patient with right eye choroidal detachment: A) conventional method of ocular ultrasound (red arrow); B) modified water bath technique (yellow arrow); and C) computed tomography image of the globe injury.

**Table 1:** Patient characteristics and findings in a case series describing use of a modified water bath technique for ocular trauma.

Age(yrs)/sex	Mechanism Of injury	Diagnosis	Ocular ultrasound finding	CT finding
Case 1 40 yr/male	Road traffic incident	Left globe injury with multiple facial fractures	Complete prolapse of the intraocular contents with extrusion of the vitreous and uveal tissue as evident from the echogenic material without any globe contour (Image 3)	Left globe rupture
Case 2 32 yr/male	Assault	Right globe injury	The irregular contour of the right globe which appears flattened due to the loss of intraocular pressure can be seen (Image 4)	Right eye globe shows loss of contour, with non-visualisation of lens likely globe rupture
Case 3 35 yr/male	Fireworks- related injury	Left globe rupture with intraocular foreign body	Ruptured globe and highly echogenic structures with posterior acoustic shadowing indicating intraocular foreign bodies. (Image 5)	Left globe rupture with intraocular foreign body
Case 4 12 yr/male	Hit by bat to the face	Left globe injury	Decrease of globe size with vitreous hemorrhage settled as a membrane at the bottom (Image 6)	Left globe injury
Case 5 42 yr/female	Assault	Right globe injury	Globe injury with choroidal detachment seen (Image 7)	Right globe injury— mildly deformed globe with choroidal detachment. Mild proptosis with thinned and stretched optic nerve. Retrobular hematoma noted along medial rectus.

*CT*, computed tomography*; yr*, year.

**Table 2:** Comparison between modified water bath technique and conventional ocular ultrasound in a case series describing their use in diagnosing ocular trauma.

Modified water bath technique	Conventional ocular ultrasound
Better quality images; hence better correlation with computed tomography	Poorer quality images
Materials easy to procure	Copious use of gel required
Less pressure transmitted to the eye when placed over the glove; hence, safer	Pressure of the probe directly over the eye posed a risk for exacerbating the ocular injury.
Increased patient comfort	Patients had a more uncomfortable experience
